# Gene-trait matching across the *Bifidobacterium longum* pan-genome reveals considerable diversity in carbohydrate catabolism among human infant strains

**DOI:** 10.1186/s12864-017-4388-9

**Published:** 2018-01-08

**Authors:** Silvia Arboleya, Francesca Bottacini, Mary O’Connell-Motherway, C. Anthony Ryan, R. Paul Ross, Douwe van Sinderen, Catherine Stanton

**Affiliations:** 1 APC Microbiome Institute, University College Cork, Cork, Ireland; 2 Teagasc Food Research Centre, Moorepark, Fermoy, Co. Cork Ireland; 3 School of Microbiology, University College Cork, Cork, Ireland; 4 Department of Neonatology, Cork University Maternity Hospital, Cork, Ireland; 5 Instituto de Productos Lácteos de Asturias (IPLA-CSIC), Paseo Río Linares, Villaviciosa, Asturias Spain

**Keywords:** *Bifidobacterium longum*, Pan-genome, Carbohydrates, Comparative genomes, Gene-trait-matching, Microbiota

## Abstract

**Background:**

*Bifidobacterium longum* is a common member of the human gut microbiota and is frequently present at high numbers in the gut microbiota of humans throughout life, thus indicative of a close symbiotic host-microbe relationship. Different mechanisms may be responsible for the high competitiveness of this taxon in its human host to allow stable establishment in the complex and dynamic intestinal microbiota environment. The objective of this study was to assess the genetic and metabolic diversity in a set of 20 *B. longum* strains, most of which had previously been isolated from infants, by performing whole genome sequencing and comparative analysis, and to analyse their carbohydrate utilization abilities using a gene-trait matching approach.

**Results:**

We analysed their pan-genome and their phylogenetic relatedness. All strains clustered in the *B. longum* ssp. *longum* phylogenetic subgroup, except for one individual strain which was found to cluster in the *B. longum* ssp. *suis* phylogenetic group. The examined strains exhibit genomic diversity, while they also varied in their sugar utilization profiles. This allowed us to perform a gene-trait matching exercise enabling the identification of five gene clusters involved in the utilization of xylo-oligosaccharides, arabinan, arabinoxylan, galactan and fucosyllactose, the latter of which is an abundant human milk oligosaccharide (HMO).

**Conclusions:**

The results showed high diversity in terms of genes and predicted glycosyl-hydrolases, as well as the ability to metabolize a large range of sugars. Moreover, we corroborate the capability of *B. longum* ssp. *longum* to metabolise HMOs. Ultimately, their intraspecific genomic diversity and the ability to consume a wide assortment of carbohydrates, ranging from plant-derived carbohydrates to HMOs, may provide an explanation for the competitive advantage and persistence of *B. longum* in the human gut microbiome.

**Electronic supplementary material:**

The online version of this article (10.1186/s12864-017-4388-9) contains supplementary material, which is available to authorized users.

## Background

The human gut microbiota harbours a complex and dynamic microbial community, which plays various functional roles to support human physiology and health [1]. This so-called “hidden organ” elicits important metabolic, structural and protective activities, and influences behaviour and brain function through the proposed “gut-brain-axis” [2, 3]. Bifidobacteria are among the first colonisers of the gut and represent common intestinal inhabitants throughout life, being purported as beneficial for the host [4–6]. Certain *Bifidobacterium* species are more frequently detected as gut commensals during early life and childhood, when the microbiota undergoes a succession to an adult-like composition after weaning [5, 7]. Bifidobacteria are Gram-positive, high G + C content, rod shaped, non-motile and non-spore-forming bacteria, belonging to the Actinobacteria phylum [8]; currently the genus *Bifidobacterium* includes 51 species and 10 subspecies, of which eight have been only recently identified (http://www.bacterio.net/bifidobacterium.html). Functional genome analysis has been vital to unravel the genetic strategies adopted by bifidobacteria to colonize the intestinal tract, a feature that appears to be facilitated by their adaptation to a glycan-rich environment [9]. More than 13% of the so-called clusters of orthologous gene (COG) families in the *Bifidobacterium* pan-genome are associated with carbohydrate metabolism [9]. Consequently, this saccharolytic genotype allows bifidobacteria to metabolize a variety of carbohydrates (host- and diet-derived substrates), which are not digested by the host’s digestive enzymes [10], to provide a competitive advantage in the complex gut environment.

Among the bifidobacteria colonizing the human gut, *Bifidobacterium longum* deserves a special mention due to its prevalence (and sometimes abundance) in both infants and adults [11, 12]. Despite the shift in microbiota composition following the transition from an exclusively milk-based diet to solid foods, it has been demonstrated that specific *B. longum* strains may persist in the intestinal microbiota over time [13, 14]; in addition, strains can be newly acquired by the adult host [15], being associated with health benefits for the host [16]. The *B. longum* taxon currently recognizes four subspecies: *longum*, *infantis*, *suis* and *suillum* [17, 18]. Isolates of the latter two subspecies are of animal origin [17, 19], while the former two are considered to be characteristic of the human gut microbiota. Genomic and functional studies have indicated that *B. longum* ssp. *infantis* is more specialized in the metabolism of milk glycans and for this reason is more adapted to the gut of a breast-feeding infant [20], being involved in the metabolism of HMOs [21]. In spite of the close genetic relatedness, *B. longum* ssp. *longum* strains are distinctly different from their *B. longum* ssp. *infantis* relatives when assessing carbohydrate utilization, with the former possessing various enzymes that are active on plant-derived oligosaccharides (while not encoding the same versatile enzymatic ability to degrade HMOs) [22, 23]. These metabolic capabilities exemplify the specific strategies followed by these two subspecies for the adaptation to a particular niche.

The correct assembly of the intestinal microbiota plays a key role in health and well-being of the host. The mechanisms of bifidobacterial establishment and persistence in the gut microbiota are far from being fully understood. However, in the particular case of *B. longum*, their diversity and capabilities to metabolize a large range of carbohydrates, considered to be the result of gene duplication and horizontal gene transfer [9], seem to make them particularly powerful microbial competitors in the complex human gut environment [24]. For these reasons, investigating metabolic abilities related to sugar fermentation and corresponding genetic functions is important to fully understand and appreciate how and which microbes can persist in the gut. In this context, the objective of this study was to explore the intraspecific genomic diversity within a pool of *B. longum* strains by performing whole genome sequencing and comparative analysis, and to analyse their carbohydrate utilization abilities using a gene-trait matching (GTM) approach.

## Results

### General genome features

In order to determine the genetic content, diversity and general characteristics of *B. longum*, especially strains previously isolated from babies (Table 1), we performed genome sequencing of 20 selected isolates in order to perform a comparative analysis against a number of publicly available *B. longum* genomes (for salient properties of these genomes see Additional file 1: Table S1). The sequencing and assembly efforts resulted in a set of unoriented contigs ranging in number from 20 to 72 per strain. In order to facilitate a coherent comparative analysis, we carried out a uniform open reading frame (ORF) prediction for all our 20 sequenced genomes in addition to the fully-sequenced *B. longum* genomes retrieved from public databases (https://www.ncbi.nlm.nih.gov/genome/genomes/183). As outlined in Table 1, the number of predicted ORFs ranged from 2189 for *B. longum* APC1503 to 1761 for *B. longum* APC1477, with an average value of 1961 ± 107 identified ORFs per genome. The average percentage G + C content was calculated to be 59.99 ± 0.20%, and ranged from 59.6% for *B. longum* APC1465 to 60.4% for *B. longum* DPC6316. The genome sizes follow a normal distribution with an average value of 2.39 Mbp, where *B. longum* APC1503 has the largest genome (2.56 Mbp) and *B. longum* APC1478 the smallest (2.22 Mbp), which is presumed to be equal or at least very close to the actual genome size as Illumina-based genome sequencing does not allow complete closure of the bacterial chromosome [25].

**Table 1:** *Bifidobacterium longum* genomes sequenced and analysed in this study

Genomes	Source of Isolation				Contigs	No. of ORFs	Genome Size (bp)	GC content (%)	No. of unique genes	Plasmids (Rep+)	Reference
	Subject	Age	Delivery	Feeding							
*B. longum* APC 1461	A	27 w	CS	BM	37	1987	2,418,994	60.0	60	No	[49]
*B. longum* APC 1462	B	1 w	NB	BM	27	1992	2,417,784	60.2	4	Yes	[49]
*B. longum* APC 1464	B	1 w	NB	BM	31	1918	2,346,522	60.0	3	Yes	[49]
*B. longum* APC 1465	C	4 w	NB	BM	57	2032	2,452,211	59.6	6	Yes	[49]
*B. longum* APC 1466	C	27 w	NB	BM	51	2026	2,419,982	59.8	0	Yes	[49]
*B. longum* APC 1468	C	27 w	NB	BM	45	1994	2,395,158	60.1	12	Yes	[49]
*B. longum* APC 1472	E	1 w	CS	BM	50	1927	2,364,041	60.1	3	Yes	[49]
*B. longum* APC 1473	F	27 w	CS	BM	39	1874	2,317,071	59.8	6	No	[49]
*B. longum* APC 1476	G	27 w	NB	BM	48	2151	2,532,540	60.0	1	Yes	[49]
*B. longum* APC 1477	H	1 w	NB	BM	24	1761	2,228,807	59.8	0	No	[49]
*B. longum* APC 1478	H	4 w	NB	BM	21	1766	2,223,352	59.8	1	No	[49]
*B. longum* APC 1480	J	4 w	NB	BM	27	2084	2,477,750	59.9	6	No	[49]
*B. longum* APC 1482	D	1 w	NB	FM	72	1899	2,337,438	60.1	17	No	[49]
*B. longum* APC 1503	D	1 w	NB	FM	39	2189	2,562,703	59.7	23	No	[49]
*B. longum* APC 1504	I	1 w	NB	BM	51	1883	2,310,288	60.2	14	No	[49]
*B. longum* DPC 6316	K	25 y	ND	ND	32	1992	2,393,969	60.4	23	Yes	[50]
*B. longum* DPC 6317	L	3 d	ND	ND	20	1987	2,448,630	60.2	8	No	[50]
*B. longum* DPC 6320	M	64 y	ND	ND	25	1852	2,330,371	59.9	9	No	[50]
*B. longum* DPC 6321	N	3 d	ND	ND	28	1941	2,382,360	59.9	24	Yes	[50]
*B. longum* DPC 6323	O	4 d	ND	ND	52	1970	2,396,960	60.2	7	Yes	[50]

*w* weeks, *y* years, *d* days, *CS* C-Section, *NB* natural birth, *BM* breast-milk, *FM* formula milk, *ND* not determined or unknown

### Comparative analyses

**Fig. 1:**
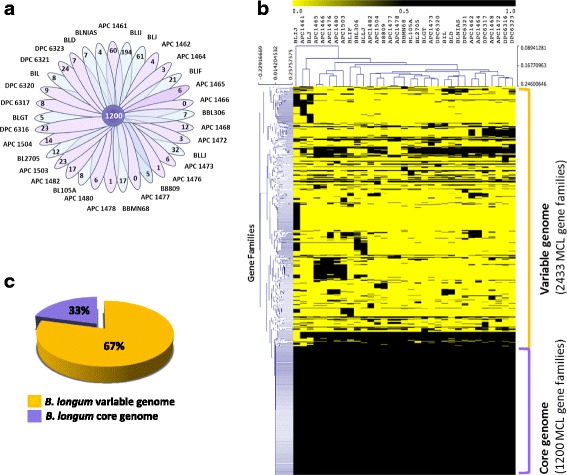
Comparative analysis of *B. longum* genomes. **a** Venn diagram displaying core gene families obtained by MCL clustering, and unique genes of *B. longum* APC/DPC strains and *B. longum* complete genomes. **b** Hierarchical clustering heatmap representing the variability of *B. longum* in terms of presence/absence of gene families. **c** Pie chart indicating the percentage of variable and core with respect to the total of gene families, resulting from the MCL clustering algorithm. **APC 1461:** *B. longum* APC 1461; **APC 1462:** *B. longum* APC 1462; **APC 1464:** *B. longum* APC 1464; **APC 1465:** *B. longum* APC 1465; **APC 1466:** *B. longum* APC 1466; **APC 1468:** *B. longum* APC 1468; **APC 1472:** *B. longum* APC 1472; **APC 1473:** *B. longum* APC 1473; **APC 1476:** *B. longum* APC 1476; **APC 1477:** *B. longum* APC 1477; **APC 1478:** *B. longum* APC 1478; **APC 1480:** *B. longum* APC 1480; **APC 1482:** *B. longum* APC 1482; **APC 1503:** *B. longum* APC 1503; **APC 1504:** *B. longum* APC 1504; **BLIJ:** *B. longum* ssp*. infantis* ATCC15697; **BLJ:** *B. longum* ssp*. longum* JDM301; **BLIF:** *B. longum* ssp*. longum* 157F; **BBL306:** *B. longum* ssp*. longum* CCUG30698; **BLLJ:** *B. longum* ssp*. longum* JCM1217; **B8809:** *B. longum* ssp*. longum* NCIMB8809; **BBMN68:** *B. longum* ssp*. longum* BBMN68; **BL105A:** *B. longum* ssp*. longum* 105A; **BL2705:** *B. longum* ssp*. longum* NCC2705; **BLGT:** *B. longum* ssp*. longum* GT15; **BIL:** *B. longum* ssp*. longum* F8; **BLD:** *B. longum* ssp*. longum* DJO10A; **BLNIAS:** *B. longum* ssp*. longum* KACC9156; **DPC 6316:** *B. longum* DPC 6316; **DPC 6317:** *B. longum* DPC 6317; **DPC 6320:** *B. longum* DPC 6320; **DPC 6321:** *B. longum* DPC 6321; **DPC 6323:** *B. longum* DPC 6323

Employing a BLASTP-mediated comparative genomic approach coupled with hierarchical clustering analysis using the MCL algorithm (see Materials and Methods), allowed us to define a pool of 1200 gene families that are common among the 20 *B. longum* APC/DPC genomes and fully sequenced genomes of *B. longum* (Additional file 1: Table S1; Fig. 1a, b), thereby representing the predicted *core genome*. Core genes are present at least once in every examined genome, and in this case account for ~33% of the total gene families obtained in our analysis (Fig. 1c). The remaining 67% (which in total constitute 2433 gene families) represent genes that make up the variable or *dispensable genome*, which consists of genes that are present in some but not all genomes (Fig. 1b, c). In line with what had been established previously [23] regarding accessory gene functions (variable genome), we observed gene families involved in the process of colonization of, and adaptation to the host environment (e.g. surface proteins, sortase-dependent pili, exopolysaccharide production, R/M systems), as well as hypothetical proteins and mobile genetic elements. Despite the fact that fragmented genome assemblies do not allow a precise estimate of the occurrence, number and chromosomal position of mobile DNA elements in genomes, a search for genes encoding plasmid replication functions (PF01051 and PF01446) revealed that at least 10 of the 20 sequenced genomes appear to contain plasmid(s) (Table 1).

Furthermore, this analysis also revealed the presence of 608 unique or strain-specific genes (i.e. genes that are present in just one of the analysed genomes), of which 227 are present in the *B. longum* APC/DPC genomes. These genes represent the presumed truly unique genes (TUGs) of each *B. longum* strain, and range between 60 in *B. longum* APC1461 to 0 in *B. longum* APC1466 and *B. longum* APC 1477 (Fig. 1a). The large percentage of genes observed within the dispensable genome is a reflection of a high level of diversity among the *B. longum* APC/DPC strains, and also with respect to publicly available genomes. Interestingly, strains isolated from the same baby generally cluster together based on hierarchical clustering analysis (Fig. 1b). For example, two pairs of *B. longum* APC/DPC strains (APC1465/APC1466 from subject C and APC1477/APC1478 from subject H) which had been isolated from samples collected at different dates, grouped together based on their gene content. In contrast, *B. longum* strains APC1462/APC1464, isolated from the same baby from samples collected in the same week exhibit clear genomic differences (Fig. 1b). Based on genome alignment, presence of SNPs and gene content it is likely that strain pair APC1477/APC1478, both isolated from the same subject H within a relatively short time frame (1 and 4 weeks), represent clonal derivatives of the same strain (we detected just 2 different genes, 24 SNPs and an average nucleotide identity of 99.9%) (Additional files 2, 3 and 4). Strains APC1465/APC1466 were isolated from subject C at an age of 4 and 27 weeks, respectively, and despite being closely related, they do contain a considerable number of genomic differences (we detected 78 different genes and an average nucleotide identity of 99.7%) (Additional files 2, 3 and 4). Interestingly, strain pair APC1462/APC1464, isolated from subject B within the same week, elicits the most striking differences (we detected 242 different genes and a lower average nucleotide identity of 99.1%). It is worth mentioning that among the genetic differences between these three strain pairs, we found functions predicted to represent mobile genetic elements (e.g. integrases, phage proteins as well as a considerable amount of hypothetical proteins) (Additional file 4). This suggests that horizontally transferred DNA plays an important role in causing genome diversity in this species.

Finally, *B. longum* APC1461 is distinct from all other sequenced *B. longum* APC/DPC strains, being the most diverse among them in terms of gene content.

### Pan-genome of *B. longum* species computation

**Fig. 2:**
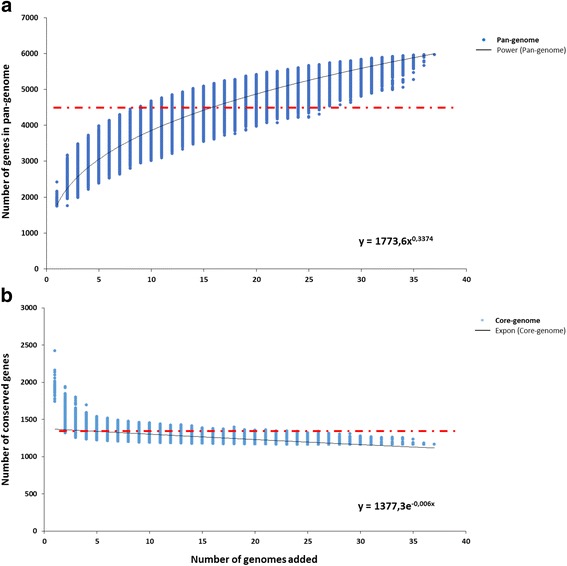
Pan-genome and core-genome of *B. longum*. **a** The pan-genome plot is represented by the accumulated number of new genes against the number of genomes added. **b** The core-genome plot is represented by the accumulated number of genes attributed to the core-genomes against the number of added genomes. The deduced mathematical function is reported. The red line represents the pan-genome and core-genome attributed to the 20 *B. longum* APC/DPC strains

In order to calculate the total gene repertoire encountered in the newly sequenced *B. longum* APC/DPC genomes and the degree of overlap and diversity with respect to other *B. longum* genomes sequenced and publicly available, a pan-genome computation was performed in two steps employing the PGAP pipeline [26]. Firstly, we calculated the pan-genome of 37 genomes, including 17 publicly available *B. longum* complete sequenced genomes (Additional file 1: Table S1) and the 20 newly sequenced *B. longum* APC/DPC genomes. This analysis generated a total of 5970 gene families for this species (Fig. 2a). The pan-genome curve was determined as the total of gene families as a function of the number of included genomes, and was shown to display an asymptotic trend with a growth rate of an average of 169 families per genome in the first 17 iterations, decreasing to an average of 55 in the final seven additions. From this calculation, after the inclusion of the 31st genome a minor increase in the pan genome size is observed. Consistent with the above, the analysis of the core-genome function shows an asymptotic trend and stabilizes after the 22nd genome iteration reaching a value of 1163 gene families at the last iteration (Fig. 2b). The trends observed in both pan-genome and core-genome functions indicate that the pan-genome calculation for the included 37 genomes is not yet fully closed, but approaching closure. For this reason, we conclude that the number of genomes analysed here provides a comprehensive and near complete overview of the gene repertoire of the *B. longum* species. Secondly, we calculated the pan-genome repertoire of just our 20 newly sequenced APC/DPC genomes. The total number of identified gene families in this case was 4309 with a core-genome size of 1295, showing an asymptotic trend consistent with what was observed for the 37 *B. longum* strains (Fig. 2). A comparison between the two-calculated pan-genomes revealed that 72% of the identified gene families are present in both, while 28% of the gene families (1698) identified in *B. longum* species are absent in the 20 newly sequenced APC/DPC genomes. As expected, analysis of the 1698 gene families that did not have representatives in the newly sequenced strains showed that 60% of these are predicted to encode hypothetical proteins, transposases, putative prophage-related proteins and generic transporters. This confirms that the high genetic diversity observed within the *B. longum* APC/DPC strains is partly attributable to what are presumed to be horizontally transferred genes.

### Phylogenetic analysis

**Fig. 3:**
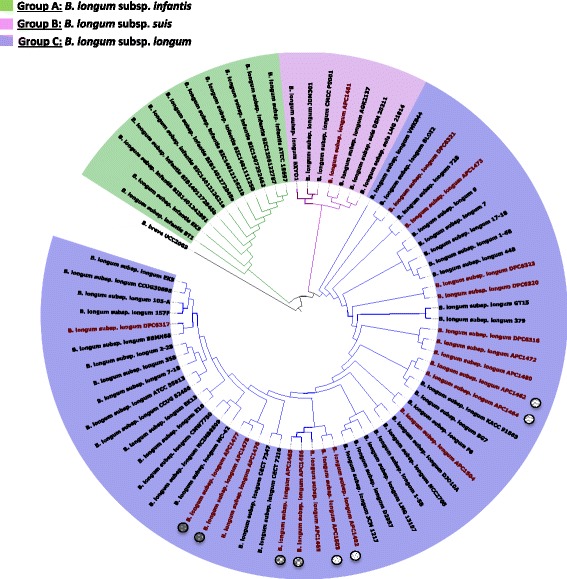
Phylogenetic analysis of *B. longum*. Phylogenetic supertree showing the relationship between 73 complete and incomplete *B. longum* strains and *B. breve* UCC 2003 as an outlier. *B. longum* APC/DPC strains are coloured in red. Grey circles with different fillings represent isolates from the same infant

For the purpose of analysing the phylogenetic relationships between the *B. longum* APC/DPC strains and other *B. longum* strains, a phylogenetic supertree was created based on sequence similarities among the gene families that constitute the core genome. The selection of families was performed based on comparative analyses and their presence in single copy between the complete *B. longum* genomes, other *B. longum* draft genomes available in the NCBI public database at the time of the study (Additional file 1: Table S1) and *Bifidobacterium breve* UCC2003 (which was selected as the outlier). The resulting phylogenetic tree (Fig. 3) reveals the presence of two major branches among the analysed members of the *B. longum* species. The first branch constitutes members of the subspecies *infantis* taxonomic group, which contains 11 representatives (Fig. 3, Group A). The second branch is split into two different clades (Fig. 3, Groups B & C). As expected, we observed that most of the genomes annotated as *B. longum* ssp. *longum* fall into the same subclade (Fig. 3, Group C), in which most of the *B. longum* APC/DPC genomes were positioned and uniformly spread, with the genome of *B. longum* APC1461 being the only exception. As expected, each of the four pairs of strains isolated from same babies, *B. longum* APC1462/APC1464, *B. longum* APC1477/APC1478, *B. longum* APC1465/APC1466 and *B. longum* APC1503/APC1482, also cluster together. Consistent with previous findings [22], the animal–derived isolates (*B. longum* ssp. *suis* LMG21814, DSM20211 and AGR2137) also cluster together (Fig. 3, Group B). Interestingly, *B. longum* APC1461 was shown to belong to this group. The genomes of *B. longum* ssp*. longum* JDM301, *B. longum* ssp. *longum* CMCCP0001 and *B. longum* ssp. *longum* BXY01 cluster together as part of a distinct sub-clade of Group B (Fig. 3, Group B), as had previously been observed [22].

### Glycosyl-hydrolase prediction in *B. longum* species

**Fig. 4:**
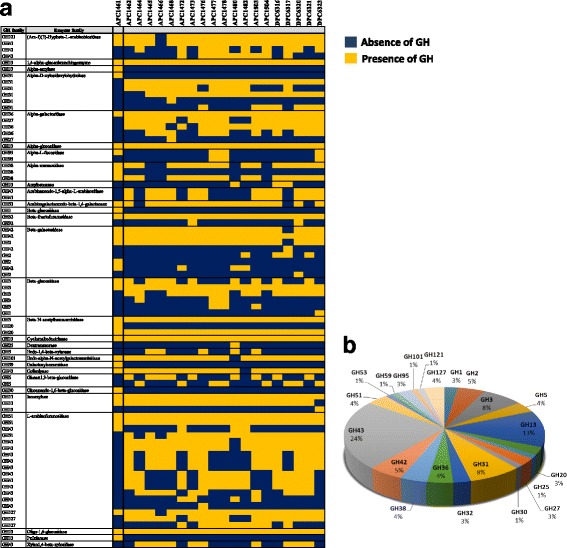
The predicted glycosyl hydrolases in *B. longum*. **a** Heatmap displaying the in silico prediction of the GH family members identified in the *B. longum* genomes. **b** Pie chart indicating the percentage of each GH family identified only in *B. longum* APC/DPC genomes

It is known that more than 13% of the genes present in bifidobacterial genomes are predicted to be involved in sugar metabolism, allowing bifidobacteria to metabolize a wide range of carbohydrates [9]. In order to assess the carbohydrate fermentation capabilities of the *B. longum* APC/DPC strains, an in silico prediction of glycosyl hydrolases (GHs) encoded in their genomes was performed using the Cazy database (Fig. 4a; Additional file 1: Table S2). This analysis revealed that the *B. longum* pan-genome contains genes encoding GHs that belong to 22 different GH families (Fig. 4b). Distributed across various strains, GHs were found that were putatively assigned to ferment plant-derived carbohydrates and classified as members of GH31 (α-D-xyloside xylohydrolase), GH32 (β-fructofuranosidase), GH43 (Ara-f)(3)-Hypβ-L-arabinobiosidase, arabinan-endo-1,5-α-L-arabinosidase, xylan-1,4-β-xylosidase, L-arabinofuranosidase), GH51 (L-arabinofuranosidase), GH121 ((Ara-f)(3)-Hypβ-L-arabinobiosidase), GH53 (arabinogalactan-endo-β-1,4-galactanase) or GH127 (L-arabinofuranosidase). Our analysis also revealed the presence of enzymes with predicted α-galactosidase (GH36, GH27), β-galactosidase (GH2, GH42), β-glucosidase (GH1, GH3) and, though at lower abundance, α-glucosidase (GH13), α-mannosidase (GH38), α-amylase (GH13) and cyclomaltodextrinase (GH13) activities (Fig. 4a; Additional file 1: Table S2). The GHs from the in silico profile allowed us to identify certain GHs that appear to be characteristic of our *B. longum* APC/DPC collection. The most distinctive profile we found was that of strain *B. longum* APC1461 (Fig. 4a). Given the phylogenomic analysis, this strain seems to belong to a separate phylogenetic group (and may represent a separate subspecies) with genomic characteristics that appear to be a mix between those of ssp. *longum* and ssp. *infantis* members. This also is reflected by the in silico GH profile which predicts a reduced number of glycolytic activities involved in hydrolysis of plant-derived saccharidic substrates, while lacking a complete HMO utilization cluster.

### In vitro bifidobacterial growth on different carbohydrate sources

**Fig. 5:**
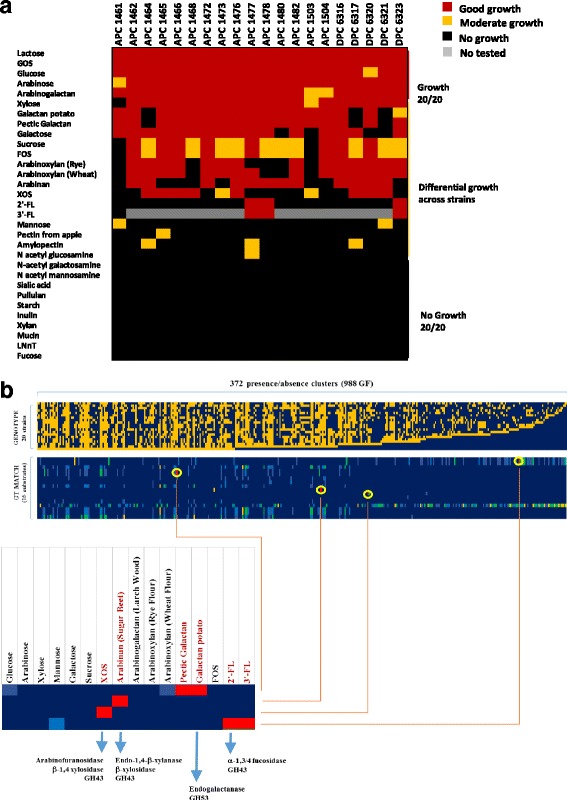
Evaluation of carbohydrate utilization by *B. longum* strains. **a** Heatmap showing the growth performance of *B. longum* APC/DPC strains on different carbon sources at 12 h. **b** Heatmap displaying the in silico gene-trait matching exercise performed based on the association between the presence/absence of GH families predicted and growth/no growth phenotype of the *B. longum* APC/DPC strains

In order to assess and validate the in silico analyses, in vitro growth assays of the *B. longum* APC/DPC strains were performed involving 31 carbohydrates as sole carbon sources (Additional file 1: Table S3; Fig. 5a; Additional file 5). In this manner, we observed that all strains were able to grow in the presence of lactose, which was therefore used as a positive control in each batch culture. All strains exhibited good growth in glucose, arabinose, arabinogalactan and GOS. In addition, just two strains, *B. longum* APC1461 and DPC6321, were capable of (limited) growth with mannose as the sole carbon source; similarly, just three strains, *B. longum* APC1464, APC1477 and DPC6317, were able to achieve (limited) growth when amylopectin was used as the sole carbon source. None of the 20 strains tested were able to metabolize N-acetyl galactosamine, N-acetyl mannosamine, sialic acid, pullulan, starch, inulin, xylan, mucin, lacto-N-neotetraose (LNnT) or fucose. The fermentation capabilities for the remainder of the tested carbohydrates were shown to be variable among the 20 strains (Fig. 5a). The APC1461 strain showed the most variable sugar utilization pattern, being the only strain that was not capable of growth on xylose. In contrast with other strains tested in this study, *B. longum* DPC6320, DPC6321, APC1480 and APC1503 did not exhibit any appreciable growth on galactose as the sole carbon source. Interestingly, *B. longum* APC1461 and APC1503 were not able to utilize fructooligosaccharides (FOS) and sucrose, whereas 10 *B. longum* strains were shown to only attain intermediate growth, while the remaining eight strains displayed good growth in the presence of these two carbohydrates (Fig. 5a). A similar trend was found in the case of utilization of two different sources of galactan: pectin galactan and potato galactan. Seventeen out of the 20 strains were shown to be able to degrade both sugars, while strains *B. longum* APC1464, DPC6317 and DPC6321 did not show those metabolic capabilities. Notably, xylo-oligosaccharides (XOS) supported growth of half of the bifidobacterial strains tested, whereas arabinan supported growth of 12 strains.

We tested two types of arabinoxylan (isolated from rye or from wheat) (Additional file 1: Table S3) and growth was observed in the presence of both carbohydrates for 14 strains, with the exception of *B. longum* APC1477, which was not able to use rye arabinoxylan though it was able to metabolize wheat arabinoxylan. Interestingly, good growth was observed for 2′-fucosyllactose (2’-FL) or 3’-FL as a unique carbon source for three out of 20 tested *B. longum* strains (Fig. 5a).

### In silico gene-trait-matching

**Fig. 6:**
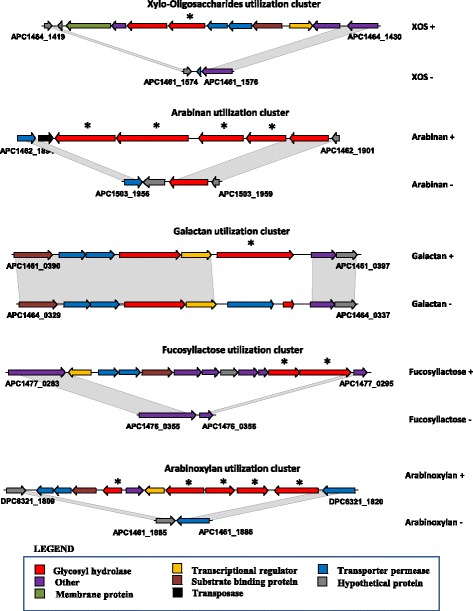
Carbohydrate utilization clusters predicted by GTM. Locus map showing gene clusters putatively involved in the utilization of various sugars by certain *B. longum* strains. Glycosyl hydrolases involved in carbohydrate utilization of each cluster are also highlighted (*)

An in silico evaluation of the role of specific genes associated with sugar metabolism found in our *B. longum* genomes was performed using a gene-trait matching (GTM) analysis based on the association between the presence or absence of gene families, and growth/no growth phenotype of the 20 *B. longum* APC/DPC strains. The analysis was performed on those carbohydrates with a differential utilization profile by the *B. longum* strains. The analyses allowed the identification of five clusters of gene families that are associated with the utilization of XOS, arabinan, arabinoxylan, galactan and fucosyllactose (Fig. 5b; Table 2; Additional file 5). Strain-specific growth on XOS corresponds with Cluster A, which consists of genes predicted to encode a 1,4-beta-xylosidase, a transporter system and a LacI-type transcriptional regulator (Table 2). Growth on arabinan was shown to correspond to Cluster B, which includes genes putatively encoding three endo-1,4-beta-xylanases and one beta-xylosidase, which is absent in all strains that are not able to grow on arabinan. The genotype/phenotype association also allowed us to identify a cluster of genes assignable to arabinoxylan utilization in 12 out of 20 strains. This cluster (Cluster C) is predicted to encode an exo-alpha-L-arabinofuranosidase II, three beta-xylosidases and one alpha-arabinofuranosidase I. Despite a partial match (<85% of phenotype/genotype concordance), 14 strains were able to metabolize arabinoxylan of which ten contain Cluster C. Moreover, three strains which were unable to grow on arabinoxylan were negative for this cluster. In contrast we observed that Cluster B may also be involved in arabinoxylan utilization, since an in silico association between the presence/absence of both clusters and the (in)ability to metabolize arabinoxylan was evident. Nine out of the 20 strains were able to grow in the presence of arabinan and arabinoxylan, and in five of these cases, both gene clusters (B and C) were present. However, when strains were shown to be unable to grow in the presence of arabinan, yet capable of growth on arabinoxylan (*B. longum* APC1466, DPC6323, APC1465, APC1473, APC1504) Cluster C was always present. Additionally, when strains were shown to metabolize arabinan yet did not grow on arabinoxylan (*B. longum* APC1478, APC1480), Cluster C was absent. Nevertheless, in the three cases of no growth observed on arabinan and arabinoxylan growth, we could only match one strain with the absence of Cluster B and Cluster C. Based on the above results, we hypothesize that both clusters are involved in the utilization of arabinoxylan, resulting in the observed ambiguous assignment. Furthermore, the observed strain-specific growth profile distribution on galactan matches with Cluster D, which contains a predicted endogalactanase. Furthermore, the lack-of-growth phenotype associated with galactan is associated with a truncation in the endogalactanase gene (resulting in a 107 amino acid-specifying pseudo-gene instead of the full gene which encodes a protein of 897 amino acids) which is believed to prevent its proper functioning. Finally, just three strains were able to utilize 2’-FL and 3’-FL as unique carbon sources and this specific growth profile allowed us to identify a gene cluster (Cluster E) encoding a predicted alpha-1,3/4-fucosidase surrounded by a LacI-like transcriptional regulator, ABC transporter system, a predicted fucose isomerase and another putative GH family 95 (Fig. 6; Table 2).

**Table 2:** Gene-trait matching with functions resulting from hierarchical clustering analysis

Carbohydrate	Gene cluster	Functions
XYLO-OLIGOSACCHARIDES (XOS)	A	Hypothetical protein
		Putative outer membrane protein
		Galactoside O-acetyltransferase
		Alpha-L-arabinofuranosidase
		*Beta-1,4-xylosidase*
		ABC transporter permease
		ABC transporter permease
		Lactose ABC transporter substrate-binding protein
		LacI family transcriptional regulator
		NADH-dependent butanol dehydrogenase 1
ARABINAN	B	*Endo-1,4-beta-xylanase*
		*Endo-1,4-beta-xylanase*
		*Beta-xylosidase*
		*Endo-1,4-beta-xylanase*
ARABINOXYLAN	C	ABC transporter, permease protein, probably fructooligosaccharide porter
		ABC transporter, permease protein, probably fructooligosaccharide porter
		ABC transporter, extracellular SBP, probably fructooligosaccharide porter
		*Exo-alpha-L-arabinofuranosidase II*
		Lipase
		LacI family transcriptional regulator
		*Beta-xylosidase*
		*Beta-xylosidase*
		*Beta-xylosidase o endo-arabinase*
		*Alpha-arabinofuranosidase I*
GALACTAN	D	Solute-binding protein of ABC transporter system for sugars galactan metabolism
		ABC transporter permease
		ABC transporter permease
		Beta-galactosidase galactan metabolism
		Transcriptional regulator LacI family galactan metabolism
		*Glycosyl hydrolases family 53 Endogalactanase galactan metabolism*
		2,5-diketo-D-gluconic acid reductase
FUCOSYLLACTOSE (FL)	E	LacI family transcriptional regulator
		putative ABC transporter permease
		ABC transporter permease
		ABC transporter substrate binding component
		Mandelate racemase/muconate lactonizing protein
		Short chain dehydrogenase
		Hypothetical protein
		Dihydrodipicolinate synthase
		Predicted fucose isomerase
		*Alpha-1,3/4-fucosidase*
		*Hypothetical protein (Glycosyl hydrolases family 95)*

Glycosyl hydrolases involved in carbohydrate utilization of each cluster are italicized

## Discussion

*Bifidobacterium longum* is one of the most abundant members of the human intestinal microbiota and is widely used as a probiotic [11, 16]. *B. longum* ssp*. longum* is commonly present at high numbers in the gut microbiota of humans throughout life, thus indicative of a close symbiotic host-microbe relationship [5, 11]. Different mechanisms may be responsible for the highly competitive nature of this taxon in its human host to allow stable establishment in the complex and dynamic intestinal microbiota environment. Genomic tools and the increased number of publicly available bifidobacterial sequences have enabled the analysis of genomic diversity and functionality in different species [9]. In this study, we carried out an analysis of the intra-species genomic diversity of 20 *B. longum* strains and we determined their capabilities to metabolize different carbohydrates.

We sequenced the genomes of 20 strains and performed a comparative genomic analysis using other publicly available *B. longum* genome sequences. Despite the fact that our genome sequencing, being based on Illumina short reads, did not permit full closure of the bacterial chromosome, we could observe that the average G + C % content and obtained genome size are in line with other *B. longum* strains sequenced [27, 28]. In contrast to observations for *B. breve* or *B. bifidum* pan-genomes [29, 30], the total predicted ORFs per genome was shown to exhibit a wide range (from 1761 to 2189), indicating that *B. longum* shows a higher level of genetic diversity than other bifidobacterial taxa, which has also been observed for the pangenome of *B. adolescentis* [31]. MCL comparative genome analysis also revealed high genomic diversity among the *B. longum* APC/DPC strains. The most diverse strain in terms of gene content was *B. longum* APC1461, which harbours the highest numbers of truly unique genes (TUGs).

The *B. longum* genomes analysed in this study allowed us to describe the pan-genome of *B. longum* species, which was defined as not fully closed but approaching a closed pan-genome, being consistent with that reported by O’Callaghan and coworkers [22]. The description of the pan-genome of other species such as *B. breve*, *B. bifidum*, and *B. animalis* ssp. *lactis* showed an essentially closed trend [29, 30, 32]. In contrast, the *B. adolescentis* pan-genome has been defined as an open pan-genome [31]. This indicates that *B. longum* shows higher genetic diversity than other bifidobacterial taxa, with the exception of *B. adolescentis*, which has also been observed to possess a wide range of predicted ORFs per genome.

The phylogenetic tree based on sequence similarity of core genes placed all but one (i.e. APC1461) of the *B. longum* APC/DPC strains in Group C of the phylogenetic super-tree, represented by members of the subspecies *longum* taxonomic group. The resulting consensus tree is in concordance with a previous one published by O’Callaghan and coworkers [22]. Group B includes representatives of the subspecies *suis* taxonomic group, which comprises strains isolated from pigs and from a calf [17, 19]. A new subspecies, *B. longum* ssp*. sullium,* was recently proposed by differentiating the subspecies *suis* taxonomic group on their ability/inability to produce urease, where the newly proposed subspecies is urease-negative [18]. However, the inclusion of this novel subspecies in the phylogenetic analysis was not possible as there is currently no representative sequenced genome available. *B. longum* APC1461 was found to carry a urease gene cluster (EC 3.5.1.5), indicating that this strain resembles subspecies *suis* rather than subspecies *sullium*. *B. longum* APC1461 is the first isolate (faecal isolate of a baby) of this subspecies to be included as a member in this clade which up until now only contains isolates of animal origin. The rest of *B. longum* APC/DPC strains were positioned clearly, yet spread uniformly across the *B. longum* clade, showing a good representation of those strains in this subspecies taxonomic group and being consistent with the genetic diversity observed.

The pan-genome analysis enabled an in silico prediction of 22 GH families involved in carbohydrate metabolism. Members of GH43 and GH13 families represented the largest proportion of all GHs predicted in APC/DPC genomes, accounting for 24% and 13%, respectively, and being consistent with previous observations for the *Bifidobacterium* genus [33]. These enzyme families are involved in the degradation of a wide range of carbohydrates, including plant-derived polysaccharides, such as amylopectin, maltodextrin, melibiose, (arabino)xylan, amylose or trehalose [34]. Other GH families such as GH31, GH51, GH53 or GH127, involved in plant-derived glycan metabolism, were commonly found in APC/DPC genomes. This enzymatic machinery is proposed to degrade the complex plant polysaccharides into mono- and oligosaccharides, which can then be taken up by ABC-type transporters (for further internal hydrolysis and metabolism). The *B. longum* APC/DPC genomes are enriched in genes that are predicted to be involved in the metabolism of plant-derived carbohydrates that are expected to be present in an adult diet [22]. However, the APC/DPC genomes also encode genes involved in the metabolism of host-derived glycans. Members of the families GH95, with fucosidase activity; GH20, hexosaminidase activity; or GH38, mannosidase activity, all exhibit a strain-specific presence in APC/DPC genomes. An endo-alpha-N-acetylgalactosaminidase (GH101), which hydrolyses the O-glycosidic bond in mucin-type O-glycan, was previously found in *B. longum* JCM1217 among others [35, 36], and is also present in all *B. longum* APC/DPC genomes with the only exception being strain APC1480. However, there was a noted absence of genes encoding sialidases or lacto-N-biosidases, which are involved in the utilization of other host-derived glycans, including particular HMOs [34].

The identification of genes or gene clusters which correlate with growth or non-growth on a particular carbohydrate was facilitated by the use of a gene-trait matching (GTM) approach, in concordance with other GTM analyses in different microorganisms [37]. We observed a strain-specific growth profile by *B. longum* APC/DPC strains on galactan, related to the presence of an endo-beta-1,4-galactanase GH53) (GalA), which has previously been reported to be involved in galactan metabolism by *B. longum* [38, 39]. Certain bifidobacterial strains/species are able to degrade xylo-oligosaccharides (XOS) and arabinoxylans (AX), complex molecules which contain arabinose and xylose substituents [40]. Both in vivo and in vitro studies have indicated the bifidogenic effect and other health benefits elicited by these potential prebiotics [40, 41]. The GTM approach applied here allowed us to identify a gene cluster (B) for arabinan utilization with a predicted endo-1,4-β-xylanase (GH5) as a key hydrolytic enzyme. Moreover, a predicted 1,4-β-xylosidase (GH43) in Cluster A is predicted to be crucial for XOS utilization, and a similar situation has previously been identified for *B. adolescentis* [42]. *B. bifidum* and *B. longum* ssp. *infantis* are known to be able to metabolize many different HMOs, and in particular the most abundant HMOs found in breast milk, fucosylated HMOs (2′-FL and 3′-FL) [43–45]. *B. longum* ssp. *longum* strains typically do not grow on 2′-FL as sole carbon source [46, 47], though a very recent study has described a *B. longum* ssp. *longum* strain capable of growing on 2′-FL or 3′-FL as the unique carbon source [48]. Interestingly, we found that three out of 20 *B. longum* APC/DPC strains exhibit vigorous growth on these sugars. This finding was supported by the prediction of a gene cluster (E) by GTM encoding an α-fucosidase, a key enzyme in HMO utilization [46] and a substrate binding component of an ABC transporter, previously found to be essential for FL intracellular utilization and being a key stable colonization factor of gut microbes in infants [47]. The positive strain found by Garrido and coworkers for 2′-FL and 3′-FL utilization showed a similar cluster of genes by transcriptomics [48], verifying our observations and the validity of our GTM approach as gene cluster predictor.

## Conclusions

In this works we studied 20 *B. longum* strains which had been isolated (mostly from infants) to explore their intra-species genomic diversity and their metabolic and genetic variation for carbohydrate consumption. According to our comparative genomic analysis, the results depict a snapshot of their substantial genetic diversity in terms of genes and predicted GHs, as well as their ability to metabolize a large range of sugars. The genomic/ phenotype of *B. longum* ssp. *longum* strains is consistent with an adult diet as exemplified by an extensive enzymatic machinery and ability of strains to degrade plant-derived carbohydrates. Furthermore, we found strains able to grow on particular HMOs. Indeed, GTM analysis predicted the key gene cluster of FL utilization, corroborating the capability of *B. longum* ssp. *longum* species to metabolise HMOs, although further biochemical analyses are required to confirm these findings. Ultimately, these observations may provide an explanation for the persistence of *B. longum* species in the gut throughout life.

## Methods

### Bacterial strains and growth conditions

The 20 bifidobacterial strains used in this study (Table 1) had previously been isolated from infant faeces [49, 50] except in two cases where the strains originate from adult faeces, and deposited in the APC Culture Collection (APC Microbiome Institute, Ireland). Bifidobacteria were routinely grown on de Man-Rogosa-Sharpe (MRS) medium (Difco Laboratories, Detroit, MI) supplemented with 0.05% *w*/*v* cysteine-HCl (Sigma, St. Louis, MO) (MRSC) and incubated at 37 °C under anaerobic conditions (10% H_2_, 10% CO_2_, and 80% N_2_) in a chamber Mac 500 (Don Whitley Scientific, West Yorkshire, UK). For solid medium, 2% (w/v) agar (Oxoid, Basingstoke, UK) was added. Prior to each DNA extraction, bacteria were sub-cultured twice and overnight cultures were used.

### Genomic DNA extraction, sequencing and data assembly

DNA was isolated by the use of a DNeasy Blood & Tissue Kit (Qiagen, Sussex, UK) following the manufacturer’s instructions. Briefly, the overnight culture was centrifuged, washed with PBS (Sigma, St. Louis, MO) and incubated for 1 h at 37 °C in an enzymatic lysis buffer with lysozyme (50 mg/ml) (Sigma, St. Louis, MO). Then, the manufacturer’s protocol was followed with the extra addition of RNAse (Sigma, St. Louis, MO). Sequencing and assembly was performed by Eurofins Genetic Services Ltd. (Ebersberg, Germany). The genomic DNA was sequenced on an Illumina MiSeq platform using chemistry v3 with paired-end sequencing. The draft genomes were de novo assembled following a pipeline that incorporates Velvet software (v1.2.10) [51] and a multi-kmer approach [52].

### Contig analysis and general feature prediction

A preliminary comparative genomic analysis (methods explained in the next section) was conducted to align the 20 *B. longum* APC/DPC genomes with all complete genomes of *B. longum* available in the NCBI public database at the time of the study (Additional file 1: Table S1), in order to select the most appropriate genome of reference for ordering contigs prior to annotation. The alignment of the 20 *B. longum* draft genome sequences was performed against a number of closely related, fully sequenced and publicly available *B. longum* genomes (Additional file 1: Table S1). The *B. longum* JDM301, *B. longum* BBMN68, *B. longum* NCIMB 8809, *B. longum* DJ010A and *B. longum* GT15 genomes were thus selected as reference genomes so as to determine presumed contig order and orientation of each draft genome. MAUVE (v2.3.1) was used to reorder contigs based on the reference genome along with Artemis software (v.14) for visualization and manual editing of the beginning of each genome at the *dnaA* gene. Prediction of putative open reading frames (ORFs) was carried out using Prodigal predictor v2.50 software (https://github.com/hyattpd/Prodigal). Identified ORFs were then automatically annotated on the basis of BLASTP analysis [53]. Functional assignment was performed against the non-redundant protein database provided by the National Centre for Biotechnology (https://www.ncbi.nlm.nih.gov). Artemis was also used for inspecting and editing, where necessary, the ORF finding outputs and the associated BLASTP results. Moreover, annotations were further refined and verified using the protein family (Pfam) (http://pfam.xfam.org) database. Glycosyl hydrolases were predicted and annotated based on similarity to the Carbohydrate-Active enZYmes (CAZy) (http://www.cazy.org/) database, Enzyme Commission number (EC) database (http://enzyme.expasy.org) using annot8r pipeline (http://www.nematodes.org/bioinformatics/annot8r) and Pfam alignments.

### Comparative genomics

An all-versus-all BLASTP alignment [53] (50% identity; e-value 1e-4 cut-off) was performed on the extracted protein sequences from each strain. The BLAST output were used as an input for the clustering into protein families sharing the same function using the Markov Cluster Algorithm (MCL) with an inflation index of 2.5, as previously described [29]. The obtained gene families were classified as belonging to either the core or to the variable genome based on their presence in either all strains or in a subset of the investigated strains, respectively. In the orthologue extraction an additional filter for paralogues was applied by selecting only those families that were shown to contain a single protein member for each genome.

### Pangenome analyses

In order to predict the possible dynamic changes of genome size at the genus level, the sizes of pan-genome, core genome and unique gene, were computed. For all the genomes used in this study, a pangenome calculation was done using PGAP [26], as previously described [29]. The ORF content of each genome was organized in functional gene clusters using the gene family (GF) method implemented in the PGAP pipeline. A pan-genome profile and a core genome profile were built using all possible BLAST combinations for each genome being sequentially added.

### Phylogenetic analyses

The supertree computation was performed from the alignment of a set of orthologous genes obtained from a BLAST-based comparative approach as indicated above, with the APC/DPC strains plus all *B. longum* genomes available in the NCBI public database at the time of writing. Each set of orthologous proteins was aligned using MUSCLE [54] and phylogenetic trees were constructed using the maximum-likelihood in PhyML [55]. The resulting consensus tree was computed using the Consense module from Phylip package v3.69 using the majority rule method (http://evolution.genetics.washington.edu/phylip.html) and phylogenetic data were submitted to TreeBASE database (http://treebase.org/treebase-web/home.html).

### Bifidobacterial growth on different carbohydrate sources and GTM associations

For evaluation of growth on the different carbohydrates sources, strains were cultured on modified MRS (mMRS) medium manually prepared to obtain the following composition: tryptone (10.0 g/L), yeast extract (5 g/L), beef extract (10.0 g/L), K_2_HPO_4_ (3.0 g/L), KH_2_PO_4_ (3.0 g/L), tri-ammonium citrate (2.0 g/L), pyruvic acid (0.2 g/L), cysteine-HCl (0.3 g/L), Tween-80 (1 mL), MgSO_4_·7H_2_O (0.575 g/L), MnSO_4_·4H_2_O (0.12 g/L), FeSO_4_·7H_2_O (0.034 g/L). Prior to autoclaving, mMRS medium was adjusted to pH 6.8.

Thirty-one carbohydrates were tested in this study (Additional file 1: Table S3). Solutions of most of them were prepared by dissolution at 5% (*w*/*v*) in distilled water and sterilized by filtration (0.45 μm). They were then added to mMRS medium at final concentration of 0.5% (*v*/v). Since certain carbohydrates do not easily dissolve in water (pullulan, starch, amylopectin, inulin, arabinoxylan (wheat and rye), pectin galactan, mucin, galactan potato, pectin apple, arabinan, arabinogalactan, xylan and amylopectin), they were added directly to mMRS medium at a final concentration of 0.5% (w/v) and autoclaved at 121 °C for 15 min.

For growth assays, *Bifidobacterium* strains were cultured at 37 °C under anaerobic conditions in 10 ml of MRSC. Afterwards they were sub-cultured twice, first at 2% (v/v) into 10 mL of fresh medium during 8 h, and then at 1% (v/v) into 10 mL of MRSC fresh medium overnight. The next day, strains were inoculated at 1% (v/v) into 10 mL of mMRS medium, previously supplemented with the soluble carbohydrates at a final concentration of 0.5% (v/v) or directly to mMRS with non-soluble carbohydrate autoclaved. Prior to inoculation, both media were supplemented with cysteine-HCl at a final concentration of 0.05%.

Growth of the bacterial strains was evaluated by optical density (OD_600 nm_) using UV-1280 spectrophotometer (Schimadzu Corporation, Kyoto, Japan). Measurements were taken manually over a 24-h period at different time points: 0, 6, 9, 12 and 24 h. mMRS supplemented with 0.05% w/v cysteine-HCl and without the addition of a carbohydrate source served as a negative control. Based on the OD_600 nm_ values at 12 h, bacterial growth was categorized as good growth (+) with an OD_600 nm_ higher than 0.5 and moderate growth (+−) with an OD_600 nm_ between 0.4 and 0.5. A cut-off value of 0.4 OD_600 nm_ was used to discriminate strains which were not able to grow in the given carbohydrates.

Following completion of the fermentation assessment of *B. longum* strains, an in silico genotype/phenotype gene-trait matching (GTM) exercise was performed, correlating the presence/absence of genes obtained from comparative analysis with an observed phenotype.

The analysis was performed on a subset of gene families obtained after exclusion of the ones present in all the strains (core-genome). A further filter was included which excluded from the analysis that fraction of genes not involved in carbon source utilization (e.g. transposases, R/M systems, CRISPR systems and prophages).

After obtaining the final number of families, a further reduction of the dataset in clusters of unique combination of occurrence was performed which allowed us to obtain the “genotype” binary matrix (values 0 for absence and 1 for presence of a gene family). This obtained matrix contained 372 clusters on the rows and 20 strains on the columns. A second binary matrix was also similarly generated containing the fermentation profile and constituting the “phenotype”. In this case, a value of 0 was assigned for OD_600_ < 0.3 and value of 1 for OD_600_ > 0.4 (taken at 12 h of growth curve). The “phenotype” binary matrix contained 16 carbohydrates on the rows and 20 strains on the columns, organized in the same order as in the genotype.

Following alignment of these two matrices, the matching percentage between each row of the two matrices and the result was represented as a heatmap. The positive matches obtained from the analysis (> 95% of match between “genotype” and “phenotype”) as well as the relative genomic surrounding regions were further inspected and compared with additional information retrieved from alternative available databases such as EC (Enzyme Classification) database [56] and PFAM (http://pfam.sanger.ac.uk) alignments, in order to further support the obtained results [29].

## Additional files

Additional file 1: Table S1. *Bifidobacterium longum* genomes publicly available used for different analysis along the study. **Table S2.** EC annotation numbers of the in silico glycosyl hydrolases predicted. **Table S3.** Carbohydrates used for in vitro assays. (DOC 152 kb)

Additional file 2: Excel file containing the SNPs identified between *Bifidobacterium longum* strain pairs APC1465 - APC1466, APC1477 - APC1478, and APC1462 - APC1464. (XLSX 1103 kb)

Additional file 3: Mauve representation containing the genome alignment of *Bifidobacterium longum* strain pairs APC1465 - APC1466, APC1477 - APC1478 and APC1462 - APC1464. (PPTX 229 kb)

Additional file 4: Excel file containing the list of MCL cluster which differ between *Bifidobacterium longum* strain pairs APC1465 - APC1466, APC1477 - APC1478 and APC1462 - APC1464. (XLSX 31 kb)

Additional file 5: Presence and absence of carbohydrate clusters predicted and growth on those carbohydrates of all the strains. (XLS 62 kb)

## Abbreviations

2’-FL: 2′-fucosyllactose
AX: Arabinoxylans
BM: Breast-milk
COG: Cluster of Orthologous Gene
CS: C-Section
D: Days
FM: Formula milk
FOS: Fructooligosaccharides
G + C: Guanine + cytosine
GHs: Glycosyl hydrolases
GTM: Gene-trait matching
HMOs: Human milk oligosaccharides
LNnT: Lacto-N-neotetraose
MCL: Markov Cluster Algorithm
NA: Not applicable
NB: Natural birth
ORF: Open reading frame
W: Weeks
XOS: Xylo-oligosaccharides
Y: Years
