# Supplementary material for: Gene-trait matching across the Bifidobacterium longum pan-genome reveals considerable diversity in carbohydrate catabolism among human infant strains
Source: BMC Genomics. 2018 Jan 8;19:33. doi: 10.1186/s12864-017-4388-9 (PMC5759876; doi:10.1186/s12864-017-4388-9)
Supplement: Supplementary file 1 — Bifidobacterium longum genomes publicly available used for different analysis along the study. Table S2. EC annotation numbers of the in silico glycosyl hydrolases predicted. Table S3. Carbohydrates used for in vitro assays. (DOC 152 kb) [file 12864_2017_4388_MOESM1_ESM.doc]

| **Table S1.** *Bifidobacterium longum* genomes publicly available used for different analysis along the study. **At the moment of the analysis.* | | | | | | | | | |
| --- | --- | --- | --- | --- | --- | --- | --- | --- | --- |
| **Genomes** | **Code** | **Used in Reference Selection** | **Used in Comparative Analysis** | **Used in the ORF prediction** | **Used in prediction of GHs** | **Used in Pangenome computation** | **Used in Phylogenetics analysis** | **Status of the genome*** | **Reference** |
| ***B. longum* JDM301** | BLJ | x | x | x | x | x | x | complete | NCBI database |
| ***B. longum* BBMN68** | BBMN68 | x | x | x | x | x | x | complete | NCBI database |
| ***B. longum* NCIMB 8809** | B8809 | x | x | x | x | x | x | complete | NCBI database |
| ***B. longum* DJO10A** | BLD | x | x | x | x | x | x | complete | NCBI database |
| ***B. longum* GT15** | BLGT | x | x | x | x | x | x | complete | NCBI database |
| ***B. longum* 105A** | BL105A | x | x | x | x | x | x | complete | NCBI database |
| ***B. longum* F8** | BIL | x | x | x | x | x | x | complete | NCBI database |
| ***B. longum* NCC 2705** | BL2705 | x | x | x | x | x | x | complete | NCBI database |
| ***B. longum* 157F** | BLIF | x | x | x | x | x | x | complete | NCBI database |
| ***B. longum* ATCC 15697** | BLIJ | x | x | x | x | x | x | complete | NCBI database |
| ***B. longum* KACC 91563** | BLNIAS | x | x | x | x | x | x | complete | NCBI database |
| ***B. longum* JCM1217** | BLLJ | x | x | x | x | x | x | complete | NCBI database |
| ***B. longum* CCUG 30698** | BBL306 | x | x | x | x | x | x | complete | NCBI database |
| ***B. longum* BXY01** | BXY01 |  |  |  |  | x | x | complete | NCBI database |
| ***B. longum* BT1** | BT1 |  |  |  |  | x |  | complete | NCBI database |
| ***B. longum* BG7** | BG7 |  |  |  |  | x | x | complete | NCBI database |
| ***B. longum* E18** | AUYD01 |  |  |  |  | x | x | complete | NCBI database |
| ***B. longum* BT1** |  |  |  |  |  |  | x | draft | NCBI database |
| ***B. longum* EK3** |  |  |  |  |  |  | x | draft | NCBI database |
| ***B. longum* BIB1401242951** |  |  |  |  |  |  | x | draft | NCBI database |
| ***B. longum* BIB1401272845b** |  |  |  |  |  |  | x | draft | NCBI database |
| ***B. longum* BIC1401212621a** |  |  |  |  |  |  | x | draft | NCBI database |
| ***B. longum* BIC1401272845a** |  |  |  |  |  |  | x | draft | NCBI database |
| ***B. longum* BIC1401212621b** |  |  |  |  |  |  | x | draft | NCBI database |
| ***B. longum* BIC1401111250** |  |  |  |  |  |  | x | draft | NCBI database |
| ***B. longum* BIC1307292462** |  |  |  |  |  |  | x | draft | NCBI database |
| ***B. longum* BIC1206122787** |  |  |  |  |  |  | x | draft | NCBI database |
| ***B. longum* CMCC P001** |  |  |  |  |  |  | x | draft | NCBI database |
| ***B. longum* AGR 2137** |  |  |  |  |  |  | x | draft | NCBI database |
| ***B. longum* DSM 20211** |  |  |  |  |  |  | x | draft | NCBI database |
| ***B. longum* LMG 21814** |  |  |  |  |  |  | x | draft | NCBI database |
| ***B. longum* VMKB44** |  |  |  |  |  |  | x | draft | NCBI database |
| ***B. longum* BLO12** |  |  |  |  |  |  | x | draft | NCBI database |
| ***B. longum* 72B** |  |  |  |  |  |  | x | draft | NCBI database |
| ***B. longum* 9** |  |  |  |  |  |  | x | draft | NCBI database |
| ***B. longum* 7** |  |  |  |  |  |  | x | draft | NCBI database |
| ***B. longum* 17-1B** |  |  |  |  |  |  | x | draft | NCBI database |
| ***B. longum* 1-6B** |  |  |  |  |  |  | x | draft | NCBI database |
| ***B. longum* 44B** |  |  |  |  |  |  | x | draft | NCBI database |
| ***B. longum* 379** |  |  |  |  |  |  | x | draft | NCBI database |
| ***B. longum* 1-5B** |  |  |  |  |  |  | x | draft | NCBI database |
| ***B. longum* LMG 13197** |  |  |  |  |  |  | x | draft | NCBI database |
| ***B. longum* D2957** |  |  |  |  |  |  | x | draft | NCBI database |
| ***B. longum* CECT 7210** |  |  |  |  |  |  | x | draft | NCBI database |
| ***B. longum* CECT 7347** |  |  |  |  |  |  | x | draft | NCBI database |
| ***B. longum* MC-42** |  |  |  |  |  |  | x | draft | NCBI database |
| ***B. longum* CMW7750** |  |  |  |  |  |  | x | draft | NCBI database |
| ***B. longum* CCUG 52486** |  |  |  |  |  |  | x | draft | NCBI database |
| ***B. longum* EK13** |  |  |  |  |  |  | x | draft | NCBI database |
| ***B. longum* ATCC 55813** |  |  |  |  |  |  | x | draft | NCBI database |
| ***B. longum* 7-1B** |  |  |  |  |  |  | x | draft | NCBI database |
| ***B. longum* 35B** |  |  |  |  |  |  | x | draft | NCBI database |
| ***B. longum* 2-2B** |  |  |  |  |  |  | x | draft | NCBI database |
| ***B. longum* EK5** |  |  |  |  |  |  | x | draft | NCBI database |

| **Table S2**: EC annotation numbers of the *in silico* glycosyl hydrolases predicted. | | |
| --- | --- | --- |
| **Enzyme family** | **GH family** | **EC numbers** |
| (Ara-f)(3)-Hypbeta-L-arabinobiosidase | GH121 | 3.2.1.187 |
| (Ara-f)(3)-Hypbeta-L-arabinobiosidase | GH43 | 3.2.1.187 |
| 1,4-alpha-glucanbranchingenzyme | GH13 | 2.4.1.18 |
| Alpha-amylase | GH13 | 3.2.1.1 |
| Alpha-D-xylosidexylohydrolase | GH31 | 3.2.1.177 |
| Alpha-galactosidase | GH36 | 3.2.1.22 |
| Alpha-galactosidase | GH27 | 3.2.1.22 |
| Alpha-glucosidase | GH13 | 3.2.1.20 |
| Alpha-L-fucosidase | GH95 | 3.2.1.51 |
| Alpha-mannosidase | GH38 | 3.2.1.24 |
| Amylosucrase | GH13 | 2.4.1.4 |
| Arabinanendo-1,5-alpha-L-arabinosidase | GH43 | 3.2.1.99 |
| Arabinogalactanendo-beta-1,4-galactanase | GH53 | 3.2.1.89 |
| Beta-glucosidase | GH1 | 3.2.1.38 |
| Beta-fructofuranosidase | GH32 | 3.2.1.26 |
| Beta-galactosidase | GH42 | 3.2.1.23 |
| Beta-galactosidase | GH2 | 3.2.1.23 |
| Beta-glucosidase | GH3 | 3.2.1.21 |
| Beta-glucosidase | GH1 | 3.2.1.21 |
| Beta-N-acetylhexosaminidase | GH3 | 3.2.1.52 |
| Beta-N-acetylhexosaminidase | GH20 | 3.2.1.52 |
| Cyclomaltodextrinase | GH13 | 3.2.1.54 |
| Dextransucrase | GH25 | 2.4.1.5 |
| Endo-1,4-beta-xylanase | GH5 | 3.2.1.8 |
| Endo-alpha-N-acetylgalactosaminidase | GH101 | 3.2.1.97 |
| Galactosylceramidase | GH59 | 3.2.1.46 |
| Gellanlyase | GH43 | 4.2.2.25 |
| Glucan1,3-beta-glucosidase | GH5 | 3.2.1.58 |
| Glucanendo-1,6-beta-glucosidase | GH30 | 3.2.1.75 |
| Isoamylase | GH13 | 3.2.1.68 |
| L-arabinofuranosidase | GH51 | 3.2.1.55 |
| L-arabinofuranosidase | GH43 | 3.2.1.55 |
| L-arabinofuranosidase | GH127 | 3.2.1.185 |
| Oligo-1,6-glucosidase | GH13 | 3.2.1.10 |
| Pullulanase | GH13 | 3.2.1.41 |
| Xylan1,4-beta-xylosidase | GH43 | 3.2.1.37 |

| **Table S3.** Carbohydrates used for *in vitro* assays. | | |
| --- | --- | --- |
| **Carbohydrates** | **Company** | **Source** |
|
| 2'-*O*-Fucosyllactose | Glycom | synthesised |
| 3'-*O*-Fucosyllactose | Glycom | synthesised |
| Amylopectin | Fluka | potato |
| Arabinan | Megazyme | Sugar Beet |
| Arabinogalactan | Megazyme | Larch Wood |
| L-Arabinose | Sigma |  |
| Arabinoxylan (rye) | Megazyme | Rye Flour |
| Arabinoxylan (wheat) | Megazyme | Wheat Flour |
| FOS | Orafti |  |
| Fucose | Glycom |  |
| Galactan | Megazyme | Potato |
| D-Galactose | Carbosynth |  |
| D-Glucose monohydrate | Sigma |  |
| Purified GOS | Friesland Camplina |  |
| Inulin | Orafti |  |
| α-Lactose monohydrate | Sigma |  |
| Lacto-N-neotretaose | Glycom |  |
| D-Mannose | Sigma |  |
| Mucin | Sigma |  |
| N-acetyl glucosamine | Carbosynth |  |
| N-acetyl mannosamine | Carbosynth |  |
| N-acetyl galactosamine | Carbosynth |  |
| Pectic Galactan | Megazyme | Potato |
| Pectin | Sigma | Apple |
| Pullulan | Hayashibara | potato |
| Sialic acid | Glycom |  |
| Starch | Sigma |  |
| Sucrose | Sigma |  |
| Xylo-oligosaccharide P95 | Longlife |  |
| Xylan | Megazyme | Beechwood |
| D-Xylose | Fluka |  |
