# Supplementary figures and images for: Gene-trait matching across the Bifidobacterium longum pan-genome reveals considerable diversity in carbohydrate catabolism among human infant strains
Source: BMC Genomics. 2018 Jan 8;19:33. doi: 10.1186/s12864-017-4388-9 (PMC5759876; doi:10.1186/s12864-017-4388-9)

## Slide 1
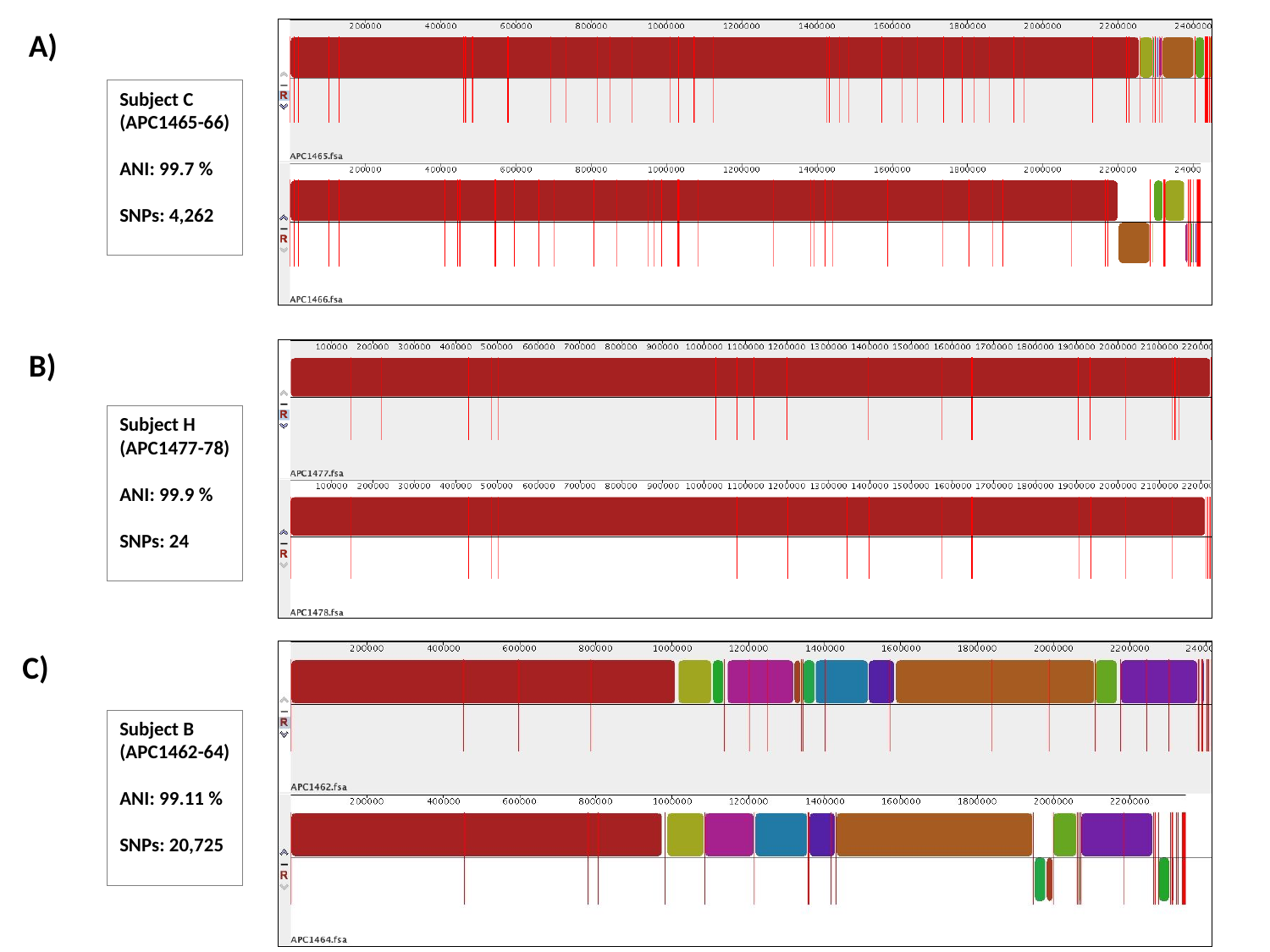

A)
Subject C
(APC1465-66)
ANI: 99.7 %
SNPs: 4,262
B)
Subject H
(APC1477-78)
ANI: 99.9 %
SNPs: 24
C)
Subject B
(APC1462-64)
ANI: 99.11 %
SNPs: 20,725

Supplement: Supplementary file 3 — Mauve representation containing the genome alignment of Bifidobacterium longum strain pairs APC1465 - APC1466, APC1477 - APC1478 and APC1462 - APC1464. (PPTX 229 kb) [file 12864_2017_4388_MOESM3_ESM.pptx]
